# Number of children ever-born and its associated factors among currently married Ethiopian women: evidence from the 2019 EMDHS using negative binomial regression

**DOI:** 10.1186/s12905-024-02883-w

**Published:** 2024-02-06

**Authors:** Mamo Nigatu Gebre

**Affiliations:** Department of Epidemiology, Faculty of Public Health, Jimma University, Jimma, Ethiopia

**Keywords:** Number of children ever-born, Currently married Ethiopian women, Evidence from the 2019 Ethiopia Mini Demographic and Health Survey

## Abstract

**Background:**

Ethiopia’s population is growing at about 2.7% annually with a fertility rate of 4.1 births per woman. However, as per the knowledge of the researcher, not enough studies have been done in Ethiopia to identify factors associated with women’s fertility levels.

**Objective:**

To assess the number of children ever born and its associated factors among currently married reproductive-age Ethiopian women.

**Method:**

Data of 5613 currently married women were extracted from the 2019 Ethiopian Mini Demographic and Health Survey (EMDHS). Stata version 14 was used for data extraction, processing, and analysis. Descriptive data were summarized using descriptive statistics. A multivariable negative binomial regression was used for the inferential analysis. Incidence rate ratio (IRR) and its 95% CI were respectively used to measure the associations and their statistical significance.

**Result:**

The median number of children ever born per currently married Ethiopian woman was 3 with an iterquarter range of 4 (1–5) children. Age of a woman at her first birth (aIRR = 0.958, 95% CI: 0.954, 0.961), being protestant (aIRR = 1.128, 95%CI: 1.068, 1.193), being Muslim (aIRR = 1.096, 95% CI: 1.043, 1.151), and being from other religious groups than Orthodox Christianity (aIRR = 1.353, 95% CI: 1.036, 1.766) are positively associated with bearing more children. On the other hands, completing primary education (aIRR = 0.664, 95% CI: 0.640,0.689), secondary education(aIRR = 0.541, 95%CI: 0.504,0.582), higher education(aIRR = 0.527, 95%CI: 0.479, 0.580), being from a richest household(aIRR = 0.899, 95%CI: 0.840, 0.962), using modern contraceptive (aIRR = 0.877, 95%CI: 0.847, 0.908), living in the Afar (aIRR = 0.785, 95%CI: 0.718,0.859), Amhara (aIRR = 0.890, 95%CI: 0.718,0.859), Gambella (aIRR = 0.894, 95%CI: 0.820,0.974), and Addid Ababa(0.845, 95%CI: 0.760,0.939) are negatively associated with bearing more children.

**Conclusion:**

Promoting women’s empowerment, encouraging women’s academic advancement, and community-based educational intervention are recommended to have optimal and decreased numbers of children.

## Background

The world’s population is more than three times larger than it was in the 1950s and is expected to peak around 2100 at a level of almost 11 billion. Most of this growth will take place in low-income and lower-middle-income countries [1, 2]. In sub-Saharan Africa, population growth rates were almost at the highest reported levels ever in 2017, when they were at 2.7% [3]. Eight countries with fast population growth: the Democratic Republic of the Congo, Egypt, Ethiopia, India, Nigeria, Pakistan, the Philippines, and the United Republic of Tanzania contribute more than half of the projected increase in global population up to 2050 [2]. The rapid global population growth that is observed since 1950 emanated from the gradual increase in average life expectancy, and the continuing high levels of fertility in several countries [1]. The world’s population growth will continue as long the fertility rate remains at its highest level [1, 2]. The global TFR decreased from 2.72 in 2000 to 2.31 in 2019, however, all countries in sub-Saharan Africa had TFRs above replacement level and accounted for 27.1% of the global live births [4].

For low and lower-middle-income countries, high fertility rates and rapid population growth exert challenges on the achievement of the sustainable development goals (SDGs); particularly, on achieving those SDGs related to health, education, and gender expediting the transition towards lower fertility [5]. For example, several low and middle-income countries including sub-Saharan Africa continued to experience high levels of adolescent fertility causing potential adverse consequences on maternal and child health [2]. The 2019 United Nations’ mortality report showed that, despite substantial reductions in maternal mortality throughout the world, disparities across regions remain large. The report also portrayed that swift action will be needed, especially in sub-Saharan Africa, to reduce child mortality to meet the target of the 2030 SDGs goals [6].

Ethiopia is among the countries with fast population growth. If Ethiopia’s population growth continues with the current momentum, its population will double in the next three decades reaching 210 million by 2060 [7]. Even though Ethiopia has recorded remarkable achievements with respect to the Sustainable Development Goals (SDGs) in recent years, the country is still sustaining a permeating multi-dimensional child poverty [8, 9]. The country’s multidimensional poverty is deep-rooted in its fast population growth [9]. A recent report depicted that 33 neonates per 1,000 live births die before their 28th birth date, and a significant proportion of children (5.88%) die before celebrating their fifth year of age [10]. Studies have shown that religion, women’s long education, and women’s empowerment are the major factors associated with women’s fertility levels [11–14]. Nevertheless, not enough studies are done in Ethiopia to identify factors associated with women’s fertility levels and the available pieces of evidence are inconclusive. The very few studies done in Ethiopia using the EDHS data had not considered the hierarchical nature of the data in the data analysis. Therefore, this study aims to assess the number of children ever born and its associated factors among currently married Ethiopian women using a nationally representative sample from the 2019 Ethiopian Mini Demographic and Health Survey.

## Methods

### Data sources

Secondary data from the 2019 Ethiopian Mini Demographic and Health Survey (EMDHS) were analyzed. The 2019 Ethiopia Mini Demographic and Health Survey (EMDHS) is the second EMDHS and the fifth DHS implemented in Ethiopia. The Ethiopian Public Health Institute (EPHI) conducted the survey in collaboration with the Central Statistical Agency (CSA) and the Federal Ministry of Health (FMoH), and other partners. The 2019 EMDHS generates data for measuring the progress of the health sector goals set under the Growth and Transformation Plan (GTP), which is closely aligned with the Sustainable Development Goals (SDG).

The survey was conducted from 21 March 2019 to 28 June 2019 based on a nationally representative sample that provided estimates at the national and regional levels and for urban and rural areas. The survey interviewed 8,855 women of reproductive age (age 15–49) from a nationally representative sample of 8,663 households. Detailed information was collected on respondents’ background characteristics, fertility determinants, marriage, awareness and use of family planning methods, child feeding practices, nutritional status of children, childhood mortality, and height and weight of children aged 0–59 months [10]. Data from 5613 currently married women were extracted for the current study as childbearing outside of marriage is not culturally tolerated in Ethiopia.

### Sample design of the 2019 Ethiopian mini demographic and health survey

The sampling frame used for the 2019 EMDHS is a frame of all census enumeration areas (EAs) created for the 2019 Ethiopia Population and Housing Census (EPHC) and conducted by the Central Statistical Agency (CSA). The census frame is a complete list of the 149,093 EAs created for the 2019 EPHC.

Administratively, Ethiopia is divided into nine geographical regions and two administrative cities. The 2019 EMDHS was a population-based cross-sectional study with a two-stage stratified cluster sampling design. Each region was stratified into urban and rural areas, yielding 21 sampling strata. Samples of EAs were selected independently in each stratum in two stages. In the first stage, a total of 305 EAs (93 in urban areas and 212 in rural areas) were selected with probability proportional to EA size (based on the 2019 EPHC frame) and with independent selection in each sampling stratum. A household listing operation was carried out in all selected EAs from January through April 2019. The resulting lists of households served as a sampling frame for the selection of households in the second stage. In the second stage of selection, a fixed number of 30 households per cluster were selected with an equal probability of systematic selection from the newly created household listing. In all selected households women aged 15–49 were interviewed using the Woman’s Questionnaire [10]. Secondary data of 5613 currently married women were extracted, weighed, and analyzed for the current study as childbearing outside of marriage is not culturally tolerated in Ethiopia. The actual number of currently married women used for the current secondary data analysis after weighting the data was 5743.

### Variables

The response variable is the number of Children ever born, whereas, the exposure variables are the age of the women, age at first childbirth, religion, highest level of education of women, Types of place of residence, Region of residence, Sex of household head, contraceptive use, and household’s wealth index. The exposure variables were selected by reviewing the literature [11–16] and based on the availability of data in the 2019 EDHS data set for the variable of interest.

### Data processing and analysis

Stata version 14 was used for data extracting, processing, and analysis. The weighting of the data was done according to the recommendation from the 2018 Guideline to DHS statistics, and all the statistical analyses were done using the weighted data [17]. Descriptive statistics were done to summarize descriptive data.

Multilevel modeling is used to analyze data that are drawn from several different levels and when the outcome is measured at the lowest level [18, 19]. The 2019 Mini Ethiopian Demographic and Health Survey was a two-stage stratified cluster sampling design that followed a hierarchical sampling technique where women are nested in households, the households are nested in enumeration areas, the enumeration areas are nested in the types of place of residence (urban or rural), and the types of place of residence is nested in the regions [10]. On the other hand, the variance (8.12) of the number of children ever born to currently married Ethiopian women is greater than its mean (3.81) indicating over-dispersion. The negative binomial regression model is used when the Poisson regression is not an appropriate model because of overdispersion [18, 20]. Therefore, to account for the hierarchical nature of the data and the overdispersion, a multilevel negative binomial regression was first fitted. In the beginning, a null model (a random intercept-only model by excluding all other explanatory variables) was fitted to test if the grouping variable at level two (region of residence) significantly affects the lower level variables to decide where or not the mixed-effect negative binomial regression model should be considered. The result of the random-intercept-only model showed that the variance component of the region of residence is not statistically significant in the random effects table (variance = 0.475, 95 CI: 0.029, 7.694). The log-likelihood ratio test of the null model also indicated that there is not enough variability between the regions to favor a mixed effect negative binomial regression (P Value > 0.05) over a single effect negative binomial regression. Hence, a multivariable negative binomial regression was fit to identify factors associated with the number of children ever born among currently married Ethiopian women. Incidence rate ratio (IRR) and its 95% CI were respectively used to measure the statistical associations between the independent variables and the number of children ever born and to measure the statistical significance.

### Ethical consideration

For the secondary data analysis, the investigator received permission from the public domain of the DHS website and reanalyzed the dataset.

## Results

### Socio-demographic, socioeconomic, and other characteristics of married reproductive-age women living in Ethiopia

The mean age of the study participants was 30.58 (SD ± 8.25) years, and 23.8% of the women were aged between 25 and 29 years of age. More than two-thirds (72.7%) of the women are rural residents, and 38.4% are Orthodox Christianity followers. More than half (51.9%) of the women have no formal education. Nearly nine in ten women (89.7%) are from the male-headed household, and 18.4% are from the poorest household. 39% of the women are from the Oromia region, and 11% of the women do not know any kind of contraceptive method. Concerning contraceptive use, 2320(40.4%) women use the modern contraceptive method, whereas, 3374 (58.7%) women do not use any type of contraceptive method (Table 1).

**Table 1:** Socio-demographic, Socioeconomic, and Other Characteristics of Currently Married Ethiopian Women, 2019

Variable	Categories	Total %	Number of Children ever born										
			Number	0	1	2	3	4	5	6	7	8	9–15
**Age**	15–19	449 (7.8)	n	264	154	27	2	1	0	0	0	0	0
			%	58.9	34.4	6.0	0.4	0.2	0.0	0.0	0.0	0.0	0.0
	20–24	929(16.2)	n	177	388	234	85	33	11	1	0	0	0
			%	19.1	41.8	25.2	9.1	3.6	1.2	0.1	0.0	0.0	0.0
	25–29	1364 (23.8)	n	90	218	389	253	182	111	73	42	4	2
			%	6.6	16.0	28.5	18.5	13.3	8.1	5.4	3.1	0.3	0.1
	30–34	1016(17.7)	n	20	42	139	181	177	169	131	101	27	29
			%	2.0	4.1	13.7	17.8	17.4	16.6	12.9	9.9	2.7	2.9
	35–39	901(15.7)	n	18	24	60	96	153	132	158	98	63	98
			%	2.0	2.7	6.7	10.7	17.0	14.7	17.6	10.9	7.0	10.9
	40–44	634(11.0)	n	10	12	13	25	72	84	84	106	88	140
			%	1.6	1.9	2.1	3.9	11.4	13.3	13.3	16.7	13.9	22.1
	45–49	449(7.8)	n	9	8	8	24	27	46	51	89	54	133
			%	2.0	1.8	1.8	5.4	6.0	10.3	11.4	19.9	12.1	29.6
**Age at first child birth**	10–14	1064	n		20	84	103	132	173	114	151	98	191
			%		1.9	7.9	9.7	12.4	16.2	10.7	14.2	9.2	17.9
	15–19	2450	n		367	403	356	327	248	266	210	103	171
			%		15.0	16.4	14.5	13.3	10.1	10.9	8.6	4.2	7.0
	20–24	1305	n		357	299	161	145	108	96	73	33	32
			%		27.4	22.9	12.3	11.1	8.3	7.4	5.6	2.5	2.5
	25–29	272	n		81	68	35	32	21	21	2	3	8
			%		29.9	25.1	12.9	11.8	7.7	7.7	0.7	1.1	3.0
	>=30	63	n		22	17	12	9	3	1	1	0	0
			%		33.8	26.2	18.5	13.8	4.6	1.5	1.5	0.0	0.0
**Type of Place of Residence**	Urban	1569(27.3)	n	221	309	309	224	132	103	70	84	38	79
			%	14.1	19.7	19.7	14.3	8.4	6.6	4.5	5.4	2.4	5.0
	Rural	4174(72.7)	n	367	538	561	443	513	451	427	352	199	323
			%	8.8	12.9	13.4	10.6	12.3	10.8	10.2	8.4	4.8	7.7
**Religion**	Orthodox	2202(38.4)	n	279	367	400	241	281	200	161	133	71	70
			%	12.7	16.7	18.1	10.9	12.7	9.1	7.3	6.0	3.2	3.2
	Catholic	30(0.5)	n	4	3	4	3	3	5	5	2	1	0
			%	13.3	10.0	13.3	10.0	10.0	16.7	16.7	6.7	3.3	0.0
	Protestant	1601(27.9)	n	116	245	214	201	140	191	143	137	86	127
			%	7.3	15.3	13.4	12.6	8.8	11.9	8.9	8.6	5.4	7.9
	Muslim	1836(32.0)	n	180	229	240	217	215	148	187	155	71	193
			%	9.8	12.5	13.1	11.8	11.7	8.1	10.2	8.5	3.9	10.5
	Traditional	62(1.1)	n	6	0	11	4	5	10	1	8	7	9
			%	9.8	0.0	18.0	6.6	8.2	16.4	1.6	13.1	11.5	14.8
	Other	11(0.2)	n	3	1	1	0	0	0	1	1	0	2
			%	33.3	11.1	11.1	0.0	0.0	0.0	11.1	11.1	0.0	22.2
**Educational Level**	No education	2979(51.9)	n	118	193	293	332	411	370	392	348	195	327
			%	4.0	6.5	9.8	11.1	13.8	12.4	13.2	11.7	6.5	11.0
	Primary	2078(36.2)	n	339	402	419	252	202	164	100	83	42	74
			%	16.3	19.4	20.2	12.1	9.7	7.9	4.8	4.0	2.0	3.6
	Secondary	453(7.9)	n	84	155	100	58	27	19	5	4	0	1
			%	18.5	34.2	22.1	12.8	6.0	4.2	1.1	0.9	0.0	0.2
	Higher	233(4.0)	n	47	96	58	25	5	1	0	1	0	0
			%	20.2	41.2	24.9	10.7	2.1	0.4	0.0	0.4	0.0	0.0
**Sex of Household Head**	Male	5153(89.7)	n	507	718	792	601	589	500	464	399	221	362
			%	9.8	13.9	15.4	11.7	11.4	9.7	9.0	7.7	4.3	7.0
	Female	590(10.3)	n	81	128	79	65	56	54	33	38	16	40
			%	13.7	21.7	13.4	11.0	9.5	9.1	5.6	6.4	2.7	6.8
**Wealth Index**	Poorest	1056(18.4)	n	76	99	142	115	141	87	136	99	61	99
			%	7.2	9.4	13.4	10.9	13.4	8.2	12.9	9.4	5.8	9.4
	Poorer	1122(19.5)	n	92	135	158	105	139	146	124	99	39	85
			%	8.2	12.0	14.1	9.4	12.4	13.0	11.1	8.8	3.5	7.6%
	Middle	1137(19.8)	n	103	145	149	136	130	144	95	101	59	74
			%	9.1	12.8	13.1	12.0	11.4	12.7	8.4	8.9	5.2	6.5
	Richer	1203(20.9)	n	125	179	182	132	106	113	106	97	51	112
			%	10.4	14.9	15.1	11.0	8.8	9.4	8.8	8.1	4.2	9.3
	Richest	1225(21.3)	n	192	288	239	179	129	65	36	39	26	31
			%	15.7	23.5	19.5	14.6	10.5	5.3	2.9	3.2	2.1	2.5
**Region**	Tigray	357(6.2)	n	28	68	64	50	38	33	30	25	10	10
			%	7.9	19.1	18.0	14.0	10.7	9.3	8.4	7.0	2.8	2.8
	Afar	63(1.1)	n	6	10	11	10	7	5	5	4	2	4
			%	9.5	15.9	17.5	15.9	11.1	7.9	7.9	6.3	3.2	6.3
	Amhara	1301(22.7)	n	160	214	222	139	174	126	97	82	43	46
			%	12.3	16.4	17.0	10.7	13.4	9.7	7.4	6.3	3.3	3.5
	Oromia	2240(39.0)	n	207	311	322	209	265	221	203	189	106	206
			%	9.2	13.9	14.4	9.3	11.8	9.9	9.1	8.4	4.7	9.2
	Somali	281(4.9)	n	33	21	27	34	25	27	33	31	12	39
			%	11.7	7.4	9.5	12.0	8.8	9.5	11.7	11.0	4.2	13.8
	Benishangul	66(1.2)	n	5	10	11	8	8	6	6	5	4	4
			%	7.4	14.7	16.2	11.8	11.8	8.8	8.8	7.4	5.9	6.0
	SNNPR	1162(20.2)	n	107	145	146	173	108	125	116	95	58	90
			%	9.2	12.5	12.5	14.9	9.3	10.7	10.0	8.2	5.0	7.7
	Gambela	24(0.4)	n	2	4	5	4	2	2	2	1	1	1
			%	8.7	17.4	21.7	17.4	8.7	8.7	8.7	4.3	4.3	4.2
	Harari	16(0.3)	n	2	3	3	2	2	2	1	1	0	1
			%	12.5	18.8	18.8	12.5	12.5	12.5	6.3	6.3	0.0	5.9
	Addis Adaba	197(3.4)	n	32	54	53	34	13	5	4	1	1	0
			%	16.2	27.4	26.9	17.3	6.6	2.5	2.0	0.5	0.5	0.0
	Dire Dawa	35(0.6)	n	6	7	6	3	3	3	1	1	1	2
			%	17.6	20.6	17.6	8.8	8.8	8.8	2.9	2.9	2.9	6.1
**Knowledge of Contraceptive Method**	Knows No Method	220 (3.8)	n	31	17	17	24	34	22	16	21	9	28
			%	14.2	7.8	7.8	11.0	15.5	10.0	7.3	9.6	4.1	12.8
	Knows Traditional Method	5(0.1)	n	0	0	1	0	0	4	0	0	0	0
			%	0.0	0.0	20.0	0.0	0.0	80.0	0.0	0.0	0.0	0.0
	Knows Modern Method	5518(96.1)	n	557	830	853	642	611	528	481	415	229	373
			%	10.1	15.0	15.5	11.6	11.1	9.6	8.7	7.5	4.1	6.8
**Contraceptive use**	No Method	3374 (58.7)	n	440	388	381	364	370	310	315	303	195	307
			%	13.0	11.5	11.3	10.8	11.0	9.2	9.3	9.0	5.8	9.1
	Traditional Methods	50 (0.9)	n	12	3	13	3	0	6	3	0	0	10
			%	24.0	6.0	26.0	6.0	0.0	12.0	6.0	0.0	0.0	20.0
	Modern Methods	2320(40.4)	n	136	455	476	300	275	238	179	133	42	85
			%	5.9	19.6	20.5	12.9	11.9	10.3	7.7	5.7	1.8	3.7
**Total**		5743(100)	n	588	846	871	666	645	554	497	437	237	402
			%	10.2	14.7	15.2	11.6	11.2	9.6	8.7	7.6	4.1	7.0

### Number of children ever born among reproductive-age women in Ethiopia

**Fig. 1:**
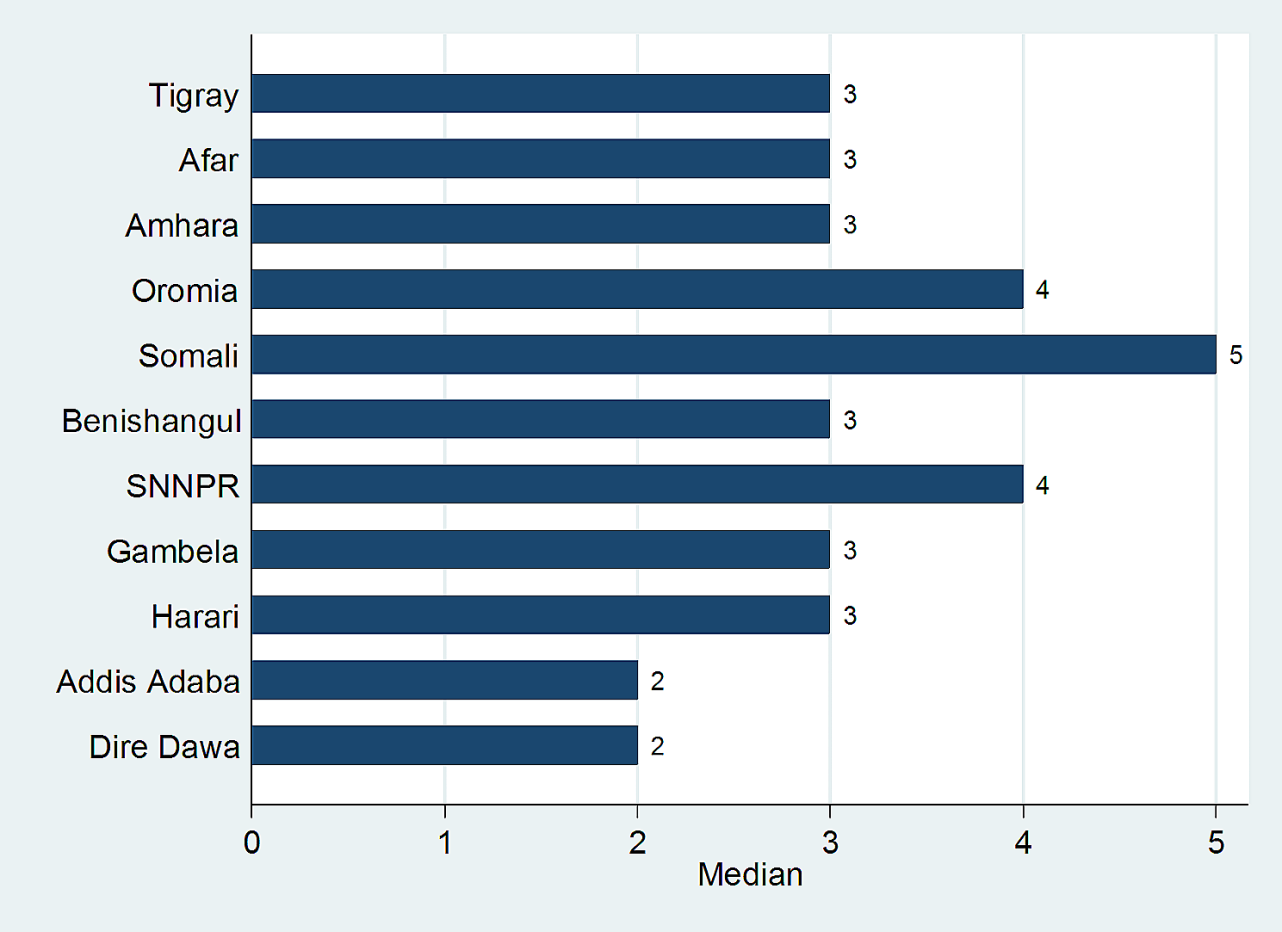
Median number of Children Ever-born Among Currently-Married Ethiopian Women, 2019

The median number of children ever born among currently married Ethiopian women was 3 with an interquartile range of 4 [1–5] children, and the mean number of children ever born was 3.81 (95%CI: 3.74, 3.89) with a standard deviation of ± 2.85 children. From the total study participants, 10.2% (*n* = 588), 14.7% (*n* = 846), 15.2% (*n* = 871), and 7.0% (*n* = 402) women had born zero, one, two, and greater than nine children respectively. Women who had born their first child at their earlier age bore more children as compared to those who had born their first child at a later age; only 3% (*n* = 8) of women who had born their first child between their 25–29 years of age have born greater than or equal to 9 children, whereas, 17.9% (*n* = 171) of women who had born their first child between their 10–14 years of age have born greater than or equal to 9 children. The number of children ever born from Ethiopian currently married reproductive-age women also varies across women’s religion categories; 10.5% (*n* = 193) of Muslim women had born greater than or equal to 9 children, whereas, only 3.2% (*n* = 70) of Orthodox Christianity follower women had born greater than or equal to 9 children. The results of the descriptive analysis also indicated that the number of children ever born consistently decreased as the women’s educational level increased; 11% (*n* = 327) of women with no education had born greater than or equal to 9 children, whereas, a maximum of 2.1% (*n* = 5) of women who had completed higher educational level have born four children. The descriptive analysis also depicted that the household’s wealth index is inversely related to the number of children ever born; only 2.5% (*n* = 31) of women from the richest household had born greater than or equal to 9 children, where 9.4% (*n* = 99) of women from the poorest household had born greater than or equal to 9 children. Modern contraceptive user women had born less number of children as compared to the women who do not use any types of contraceptive; 9.1% (*n* = 307) of non-contraceptive user women had born more than or equal to 9 children where only 3.7% (*n* = 85) of modern contraceptive women had born greater than or equal to nine children (Table 1). The median number of children ever born among the currently married women in Ethiopia varies across the geographical regions ranging from 2 children per woman in Addis Ababa and Dire Dawa to 5 children per woman in the Somali region (Fig. 1).

### Model selection criteria and test of overdispersion

The result of the random intercept-only model of the multilevel negative binomial regression indicated that the variance components of the upper-level grouping variable (region of residence) is not statistically significant to consider the multilevel negative binomial regression (Variance = 0.475, 95% CI:0.030,7.695) (Table 2). Besides, the log-likelihood ratios test also depicted that there is not enough variability between the regions to favor the multilevel negative binomial regression over negative binomial regression (p-value > 0.05). Therefore, the negative binomial regression model is considered for the final analysis to identify factors associated with the number of children ever born among currently married Ethiopian women. After selecting the model, again, the test of overdispersion of the variance was done to decide between Poisson and negative binomial regression. According to the test of overdispersion of the variance in the negative binomial model, the null hypothesis (H_o_) proposes that the mean is equal to its variance (α = 0, there is no overdispersion) supporting the Poisson regression over the negative binomial regression, whereas, the alternative hypothesis (H_A_) argues that there is overdispersion (α > 0) in the data set supporting the negative binomial regression. The result of the negative binomial regression indicated that there is overdispersion in the data set (α = 0.031) (Table 3). The log-likelihood ratio test also indicated that alpha is greater than zero (Log Chi^2^ (01) = 42.07, p-value < 0.001) reaffirming the existence of overdispersion which favors the use of negative binomial regression over the Poisson regression model (Table 2). Hence, the overall model analysis indicated that negative binomial regression is a more appropriate model than both Poisson and multilevel negative binomial regressions, and it was used for the final data analysis.

**Table 2:** Parameters of multilevel and single level negative binomial regression

**Parameters of the random intercept only model of the multilevel negative binomial regression**	
Region level variance (95%CI)	0.475(0.030,7.695)
Median incident rate ratio (95% CI)	3.748(3.671,3.827)
AIC	26075.32
BIC	26095.22
Log likelihood	-13034.658
**Information criteria and the overdispersion test of the multivariable single level negative binomial regression**	
Information criteria	Multivariable Negative binomial Regression
AIC	20943.72
BIC	21145.86
Log likelyhood test of α = 0	Log Chi2(01) = 42.07, p value < 0.001

**Table 3:** Factors associated with the number of CEB in Ethiopia

Total number of Children ever born	aIRR	Std. Err.	z	P > z	[95% Conf. Interval of aIRR]	
					Lower	Upper
**Age at first child birth**	0.958	0.002	-22.08	0.000	0.954	0.961*
**Types of place of residence**						
Urban	1					
Rural	0.963	0.027	-1.39	0.164	0.912	1.016
**Religion**						
Orthodox	1				1	
Catholic	1.055	0.100	0.57	0.569	0.876	1.271
Protestant	1.128	0.032	4.28	0.000	1.068	1.193*
Muslim	1.096	0.028	3.64	0.000	1.043	1.151*
Traditional	1.065	0.084	0.79	0.429	0.912	1.243
Other	1.353	0.019	2.22	0.027	1.036	1.766*
**Highest educational level**						
No education	1					
Primary	0.664	0.013	-21.79	0.000	0.640	0.689*
Secondary	0.541	0.020	-16.85	0.000	0.504	0.582*
Higher	0.527	0.026	-13.11	0.000	0.479	0.580*
**Sex of household head**						
Male	1					
Female	0.976	0.022	-1.11	0.265	0.934	1.019
**Wealth Index**						
Poorest	1					
Poorer	0.981	0.024	-0.78	0.437	0.936	1.030
Middle	0.995	0.0252	-0.21	0.834	0.947	1.045
Richer	0.989	0.026	-0.44	0.659	0.940	1.040
Richest	0.899	0.031	-3.06	0.002	0.840	0.962*
**KnowledgeContraceptive**						
Knows no method	1					
Knows traditional methods	0.872	0.175	-0.68	0.495	0.588	1.292
knows modern methods	1.006	0.003	0.21	0.837	0.947	1.069
**ContraceptiveUse**						
No method	1				1	
Traditional Methods	1.069	0.093	0.76	0.447	0.901	1.266
Modern Methods	0.877	0.016	-7.43	0.000	0.847	0.908*
**Region**						
Tigray	1					
Afar	0.785	0.036	-5.30	0.000	0.718	0.859*
Amhara	0.890	0.032	-3.23	0.001	0.830	0.955*
Oromia	1.017	0.039	0.44	0.660	0.943	1.097
Somali	1.043	0.048	0.91	0.362	0.953	1.141
Benishangul	0.950	0.0374	-1.31	0.192	0.880	1.027
SNNPR	1.006	0.040	0.13	0.899	0.930	1.087
Gambela	0.894	0.040	-2.57	0.010	0.820	0.974*
Harari	0.934	0.042	-1.50	0.134	0.856	1.021
Addis Adaba	0.845	0.046	-3.13	0.002	0.760	0.939*
Dire Dawa	0.957	0.044	-0.96	0.336	0.875	1.047
_cons	11.463	0.727	38.44	0.000	10.122	12.981
/lnalpha	-3.462	0.170			-3.796	-3.129
alpha	0.031	0.005			0.023	0.044

1 = reference, *=statistically significant

### Factors associated with number of children ever born among currently married reproductive-age Ethiopian women

A multivariable negative binomial regression was fitted to identify factors independently associated with the number of children ever born among reproductive-age Ethiopian women. Accordingly, the age of a woman at her first childbirth, religion of a woman, level of education of a woman, s, wealth index of a household, contraceptive use, and region of residence are independently associated with the number of children ever born among the currently married Ethiopian women. The result showed that the number of children ever born decreases by 4.2% (aIRR = 0.958, 95%CI: 0.954,0.961) as a woman’s age at first childbirth increases by 1 year controlling for all other variables. A protestant Christianity follower woman, a Muslim woman, or a woman who follows another religion is 12.8% (aIRR = 1.128, 95%CI:1.068,1.193), 9.6% (aIRR = 1.096, 95% CI:1.043,1.151), and 35.3% (aIRR = 1.353, 95% CI:1.036,1.766) more likely to have more number of children ever born as compared to an Orthodox Christianity follower woman controlled for all other variables. A woman who completed primary education, secondary education, or higher education is 33.6% (aIRR = 0.664, 95% CI:0.640,0.689), 45.9% (aIRR = 0.541, 95%CI:0.504,0.582), and 47.3% (aIRR = 0.527, 95%CI: 0.479,0.580) less likely to have more number of children ever born as compared to a woman with no education when controlling for all confounding variables. A woman from the richest household is 10.1% (aIRR = 0.899, 95%CI: 0.840, 0.962)) less likely to have more children ever born as compared to a woman from the poorest household. A modern contraceptive method user woman is 12.3% (aIRR = 0.877, 95%CI: 0.847, 0.908) less likely to have more number of children ever born as compared to a woman who does not use any type of contraceptive method controlling for all other variables. A woman who resides in Afar, Amhara, Gambella, and Addis Ababa region is 21.5% (aIRR = 0.785, 95%CI: 0.718,0.859), 11% (aIRR = 0.890, 95%CI: 0.830,0.955), 10.6% (aIRR = 0.894, 95%CI: 0.820,0.974), and 15.5% (aIRR = 0.845, 95%CI: 0.760,0.939) less likely to have more number of children ever born as compared to a woman who resides in the Tigray region of Ethiopia controlled for all other variables(Table 3).

## Discussion

The mean number of children ever born among currently married Ethiopian women was 3.81 (95%CI: 3.74, 3.89) with a standard deviation of ± 2.85 years. This result is similar to the previous study done in Ethiopia [14]. The study showed that the average number of children born per woman is below the 2019 EMDHS report which was 6.7 children per married woman [10]. The difference is due to the use of married women of different age groups for the report: the current study used married women of age between 15 and 49 years, whereas, the Mini Demographic Health Survey Report used married women of age between 45 and 49 years. Naturally, the probability of bearing more children increases for a woman in a marital union until she reaches her menopausal age. The study also indicated that the median number of children ever born per married Ethiopian woman varies across the geographical regions of the country ranging from 2 children per woman in the Capital city of the country, Addis Ababa and Dire Dawa to 5 children in the Somali Region. The study showed that the age of a woman at her first childbirth, the religion of a woman, the level of education of a woman, the wealth index of a household, the contraceptive utilization, and the region of residence are independently associated with the number of children ever born among the currently married Ethiopian women.

The likelihood of having more children ever born decreases by 4.2% as the woman’s age at her first childbirth increases by one year. This finding is consistent with the results of the study done in Bangladesh [11] where women who had born their first child at an earlier age are more likely to bear more children. On the other hand, the study done in Ethiopia on an ideal number of children among Ethiopian women using the 2016 EDHS showed that women who bear their first child at a later age are less likely to have more children as compared with women who bear their first child at their earlier age [16].

A protestant Christian follower woman, a Muslim woman, or a woman who follows another religion is 14.9%, 23.8%, and 56.1% more likely to have more children ever born as compared to an Orthodox Christian follower woman. This finding is similar to the study done in Bangladesh using the secondary data analysis of BDHS where Muslim women are 39.37% more likely to have more children ever born as compared to non-Muslim women [15]. The finding is also concordant with the result of the previous study done in Ethiopia [13] where a Protestant woman, a Muslim woman, and a woman following another religion than Orthodox Christianity had an increased likelihood of having more children as compared to a woman following Orthodox Christianity. These differences could be because of the differences in doctrinal and dogmas across the different religious groups. The study done in western Ethiopia showed that women are influenced by their religion not to use modern contraceptives; Muslim women are 65% less likely to utilize modern contraceptives as compared to Orthodox Christian women [21]. The current study also revealed that contraceptive-user women are less likely to bear a higher number of children as compared to non-contraceptive women.

A woman who completed primary education, secondary education, or higher education is 33.6%, 45.9%, and 47.3% less likely to have more children ever born respectively as compared to a woman with no education. This finding supports the results from the studies done in Bangladesh [11], Oman [12], and Ethiopia [13, 14] where the women’s level of education was inversely associated with women’s likelihood of having more children. The study done in 141 countries including the Sub-Saharan African countries indicated that fertility rate is inversely correlated with educational level [22]. Studies have witnessed that women’s educational advancement brings about delayed marriage, delayed childbearing, small family size, and women empowerment [23–26].

A woman from the richest household is 10.1% less likely to have more children as compared to a woman from the poorest household. This finding is in line with findings from the previous studies [12, 27–29] where the wealth index was negatively associated with the likelihood of having more children. This might be due to the reason that women from low-income households are less likely to advance in education and more likely to bear a higher number of children. The 2019 EMDHS report showed that women in the highest wealth quintile (12%) are more likely than women in the lowest wealth quintile (< 1%) to have more than a secondary education, and women with no education are more likely to live in poverty. Besides, the report also indicated that 59% of women in the lowest wealth quintile have no education, compared with 24% of women in the highest quintile. The current study and many more aforementioned studies portrayed that women’s long education is associated with a lower number of children and optimal family size.

As expected, contraceptive utilization is also significantly associated with number of children ever born. A woman who uses modern contraceptive methods is 12.3% less likely to have a higher number of children ever born as compared to a woman who does not use any type of contraceptive method. This finding is concordant with the results of the study done in Oman [12] and Nepal [30] where contraceptive-user women are less likely to have a higher number of children as compared to non-contraceptive-user women. The result is also in line with the two different studies done in Ethiopia using the 2016 EDHS where contraceptive-user women are less likely to bear more children [14, 16].

The region of residence is also independently and significantly associated with the number of children ever born. A woman residing in Afar, Amhara, Gambella, and Addis Ababa region is 21.5%, 11%, 10.6%, and 15.5% less likely to have more children ever born as compared to a woman who resides in the Tigray region of Ethiopia respectively controlled for all other variables. This finding is consistent with the previous study done in Ethiopia using the 2016 EDHS where women living in the Afar, Amhara, Gambella, and Dire Dawa regions had a smaller number of children ever born compared to the women residing in the Tigray region.

The current study has its strengths and limitations. The study used the data from the nationally representative EMDHS with a sufficiently large sample size, therefore, it has high power to yield valid and dependable results. On the other hand, the 2019 EMDHS lacks data on the sociocultural factors; hence, I did not assess the association between the number of children ever-born and its associated sociocultural factors among the currently married Ethiopian women. Besides, as the data were collected at a single time, the current study does not show the temporality between the number of children ever born and its associated factors among currently married Ethiopian women, and the results should be used cautiously.

## Conclusion

The mean number of children ever born among currently married Ethiopian women was 3.81 with a standard deviation of ± 2.85 years. The age of a woman at first childbirth, religion of a woman, level of education of a woman, wealth index of a household, contraceptive use, and region of residence are independently associated with the number of children ever born among the currently married Ethiopian women. Women empowerment with economy and education is recommended to have a lower number of children giving due focus to non-Orthodox religion group women.

## Data Availability

The datasets used in the current study are openly accessed and are freely available from a public domain MEASUREDHS website. https://dhsprogram.com/data/dataset_admin/login_main.cfm?CFID=10106966&CFTOKEN=a531226989613ac0-7B7AD8A7-E45D-2B2E-C20F5CFFAB6B0B60.

## Abbreviations

aIRR: Adjusted incident rate ratio
AIC: Akaike Information Criterion
BIC: Bayesian Information Criterion
CI: Confidence Interval
CSA: Central Statistical Agency
DHS: Demographic and Health Survey
EA: Enumeration Area
EMDHS: Ethiopia Mini Demographic and Health Survey
EPHC: Ethiopia Population and Housing Census
EPHI: Ethiopia Public Health Institute
FMoH: Federal Ministry of Health
GTP: Growth and Transformation Plan
IRR: Incidence Rate Ratio
SDG: Sustainable Development Goal
TFR: Total Fertility Rate
UN: United Nation
WASH: Water, Sanitation and Hygiene
